# Comparison of Efficacy and Safety Between Immunotherapy and Docetaxel Monotherapy in NSCLC Patients

**DOI:** 10.3389/fonc.2022.883514

**Published:** 2022-08-10

**Authors:** Wenchao Yang, Bixia Xuan, Mengqi Chen, Xiaofang Li, Jiana He, Haiyan Si, Yefei Zhang

**Affiliations:** ^1^ Department of Pharmacy, Traditional Chinese Medical Hospital of Zhuji, Zhuji, China; ^2^ Department of Gastroenterology, Traditional Chinese Medical Hospital of Zhuji, Zhuji, China; ^3^ Department of Pharmacy, Zhuji People’s Hospital of Zhejiang Province, Zhuji, China

**Keywords:** immune checkpoint inhibitors, docetaxel, non small cell lung cancer, overall survival, progression free survival, security

## Abstract

**Objective:**

Meta analysis was used to compare the efficacy and safety of immune checkpoint inhibitor and docetaxel in the treatment of non-small cell lung cancer.

**Methods:**

CNKI, CBM, PubMed, EMBASE, Cochrane Library, web of science and other databases were searched by computer, and the randomized controlled trials of immune checkpoint inhibitors and docetaxel in the treatment of NSCLC published as of February 2022 were collected. Two researchers searched independently, screened the literature and extracted the data according to the nanodischarge criteria, and used Revman5.4. The included studies were statistically analyzed, and publication bias was analyzed with Egger test in Stata12.

**Results:**

A total of 8 RCTs were included, including 2444 cases treated with immune checkpoint inhibitors and 2097 cases treated with docetaxel. Compared with docetaxel, the overall survival (HR = 1.40, 95%CI: 1.30-1.50, P < 0.00001) and progression free survival (HR = 1.22, 95%CI: 1.13-1.32, P < 0.00001) of NSCLC treated with ICIs were longer. The risk ratio of any grade of adverse reactions (HR = 0.41, 95%CI: 0.32-0.52, P < 0.00001) and above grade III adverse reactions (HR = 0.27, 95%CI: 0.18-0.41, P < 0.00001) in the treatment of NSCLC with ICIs was lower. There was no publication bias in Egger test.

**Conclusion:**

Compared with docetaxel, immune checkpoint inhibitor treatment can improve the clinical efficacy of NSCLC patients and has a lower incidence of adverse reactions. This treatment may be a promising treatment for NSCLC patients.

## Introduction

Lung cancer is one of the most common malignant tumors in China. Its incidence rate and mortality rate are the first in all tumors. Lung cancer is mainly divided into small cell lung cancer (SCLC) and non-small cell lung cancer (NSCLC), of which NSCLC accounts for 85%. Non-small cell lung cancer includes squamous cell carcinoma, adenocarcinoma and large cell carcinoma. The etiology of lung cancer is complex. At present, it is considered that it is mainly related to smoking, air pollution, occupational factors and changes in molecular genetics. The early symptoms of lung cancer are not obvious. Later, there are often symptoms such as cough, blood in sputum, chest pain and so on (1, 2). Due to the limited diagnostic tools currently used, 75% of patients were found to be in advanced stage. The prognosis of most patients is poor. Based on the stage of the disease at the time of diagnosis, the 5-year survival rate of patients is 4% - 17% (3). At present, docetaxel chemotherapy is one of the most commonly used second-line treatments for NSCLC (2, 3). Its mechanism is to increase the polymerization of tubulin, and then inhibit the depolymerization of microtubules, and thus inhibit the division and growth of tumor cells (4). However, docetaxel has poor efficacy and high toxicity in the treatment of NSCLC. Therefore, it is necessary to explore new treatment methods to prolong the survival time and improve the quality of life of patients.

In recent years, with the rapid development of tumor immunology, immunotherapy has become another new tumor treatment method besides surgery, chemotherapy and radiotherapy. Great breakthroughs have been made in the research of immune checkpoint inhibitors, and the role of inhibitory antibodies in clinical treatment trials of malignant tumors has also been recognized. PD-1 is a cell surface receptor, which is highly expressed on activated T cells and is considered to be a marker of T cell failure. It can regulate the activity of T cells, activate the apoptosis of tumor specific T cells and inhibit the apoptosis of regulatory T cells, so as to inhibit immune response and promote self tolerance (5). PD-L1 is expressed on some types of tumor cells and antigen-presenting cells and is considered to be a co suppressor of immune response. It can be bind to PD-1, activate PD-1/PD-L1 pathway, inhibit downstream signal transduction and T cell biological function, lead to tumor specific T cell failure and apoptosis, and make tumor cells escape immune surveillance (6). PD-1/PD-L1 pathway induces and maintains immune tolerance in tumor microenvironment and promotes tumor development. In this study, randomized controlled trials (RCTs) of ICIs and docetaxel monotherapy in the treatment of NSCLC were searched and efficacy and safety were evaluated by meta-analysis. The results obtained can provide a reference for clinical treatment.

## Methods and Materials

### Search Strategy

We used computers to search PubMed, EMBASE, Cochrane Library, Web of science database, etc. Chinese search terms: immune checkpoint inhibitor, docetaxel, non-small cell lung cancer, randomized controlled trial; English search terms: ICIs, docetaxel, non small cell lung cancer, NSCLC, randomized controlled trials, RCTs. The search deadline is February 2022.

### Study Selection

Inclusion criteria: ① Literature: retrospective study, prospective study; ② The subjects were patients with NSCLC diagnosed by clinicopathological examination; ③ Intervention measures: patients in the experimental group treated with ICI monotherapy and patients in the control group treated with docetaxel monotherapy; ④ The primary clinical outcome measures were overall survival (OS) and progression free survival (PFS). The secondary outcome measures were adverse reactions at any level and adverse reactions above grade 3.

Exclusion criteria: ① repeatedly published literature; ② Documents that cannot obtain original data or contact the author to obtain the original text; ③ Abstract, review, meta-analysis, case report and animal experiment; ④ Non Chinese and English literature.

### Literature Screening and Data Extraction

Two researchers independently read the initial literature titles and abstracts according to the inclusion and exclusion criteria, screened the literature that may meet the inclusion criteria by reading the full text, and extracted the data according to the pre-designed table, including the first author, year of publication, number of patients, OS, PFS and adverse reactions. In case of differences, the two researchers shall discuss and solve them.

### Bias Risk Assessment

We assessed the quality of inclusion in clinical randomized controlled trials, met the criteria proposed in the Cochrane manual for systematic evaluation of interventions (5.1.0), and evaluated the generation of random sequences, allocation concealment, blinding of participants, blinding of outcome evaluation, and incomplete outcome data to ensure a low incidence of bias.

### Statistical Analysis

We use Revman5.4 software to analyze the data of the included studies. Relative risk ratios (RR) and 95% confidence interval (95%CI) were used as effect indexes for counting data, and the difference was statistically significant (P < 0.05). I^2^ is used to evaluate the heterogeneity. If the heterogeneity test result I^2^ is less than 50%, it means that there is no statistical heterogeneity among the research results, and the fixed effect model is used; If the heterogeneity test result I^2^ > 50%, analyze the source of heterogeneity. If the heterogeneity still exists, select the random effect model to estimate the combined effect.

## Results

### Literature Search and Screening

**Figure 1:**
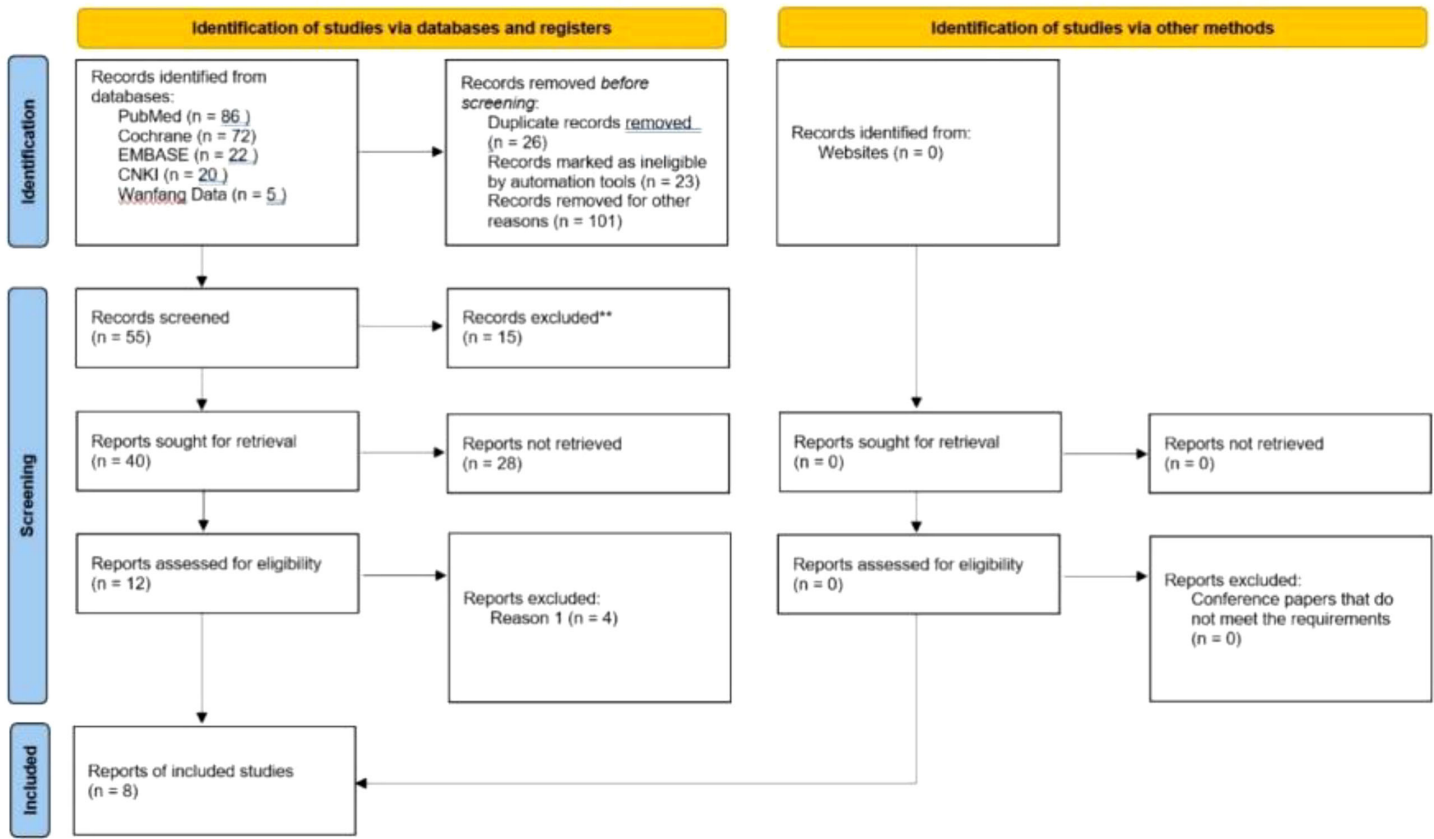
Literature screening process and results.

205 literatures (including 86 PubMed, 72 Cochrane, 22 Embase, 20 CNKI, and Wanfang VIP5) were searched by computer, and 55 were selected according to the title and abstract. After full-text analysis and evaluation, 47 literatures with abnormal data, incomplete information or unavailable due to non comparative research were excluded, and finally 8 (7–14) literatures were included for systematic evaluation and meta-analysis. The process of literature screening is shown in **Figure 1**. Among them, 2444 patients were treated with ICIs monotherapy and 2097 patients were treated with docetaxel monotherapy. **Table 1** summarizes the basic characteristics and main evaluation indicators of the included research.

**Table 1:** Basic characteristics of included studies and main evaluation indicators (7–14).

First Author	Year	Clinical trial number	Phase	Type of Cancer	NO. of Patients with ICI	NO. of Patients with Docetaxel	HR for OS[95% CI]	p-Value for OS	HR for PFS[95% CI]	p-Value for PFS
Leora Horn (8)	2017	NCT01673867	III	NSCLC	427	427	0.72[0.62,0.84]	0.001	NA	NA
H. Borghaei (9)	2015	NCT01673867	III	NSCLC	292	290	0.73[0.59,0.89]	0.002	0.92[0.77,1.11]	0.39
Julie Brahmer (10)	2015	NCT01642004	III	NSCLC	135	137	0.59[0.44,0.79]	0.001	0.62[0.47,0.81]	0.001
Shun Lu (11)	2021	NCT02613507	III	NSCLC	338	166	0.75[0.61,0.93]	0.001	0.79[0.65,0.98]	0.001
Yi-Long Wu (12)	2019	NCT02613507	III	NSCLC	338	166	0.68[0.52,0.90]	0.0006	0.77[0.62,0.95]	0.0147
Roy S Herbst (13)	2015	NCT01905657	III	NSCLC	345	343	0.71[0.58,0.88]	0.0008	0.88[0.74,1.05]	0.07
Louis Fehrenbacher (14)	2016	NCT01903993	II	NSCLC	144	143	0.73[0.53,0.99]	0.04	NA	NA
Achim Rittmeyer (15)	2016	NCT02008227	III	NSCLC	425	425	0.73[0.62,0.87]	0.0003	NA	NA

ICIs, immune checkpoint inhibitors; NSCLC, non-small cell lung cancer; HR, Hazard ratio; NA, Not available; PFS, Progression free survival; OS, Overall survival.

### Quality Assessment Results

**Figure 2:**
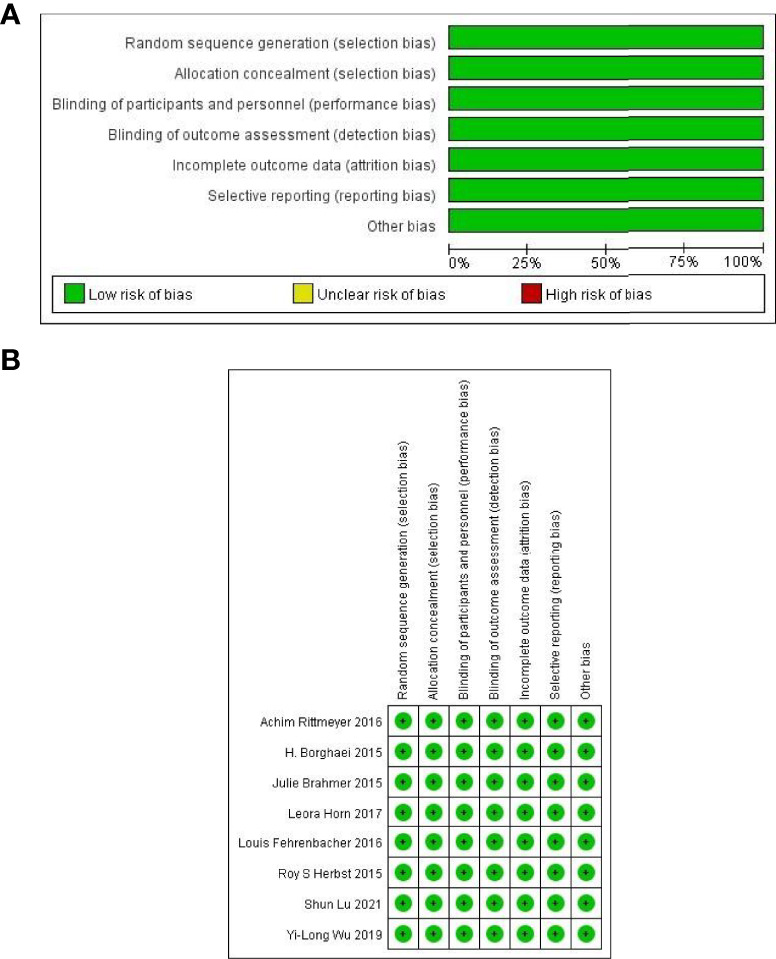
**(A, B)** Evaluation results of methodology quality of included studies (7–14).

The quality assessment results are shown in **Figures 2A, B**. All included studies had a low risk of bias.

### Meta Analysis Results of Effectiveness of ICIs and Docetaxel

**Figure 3:**
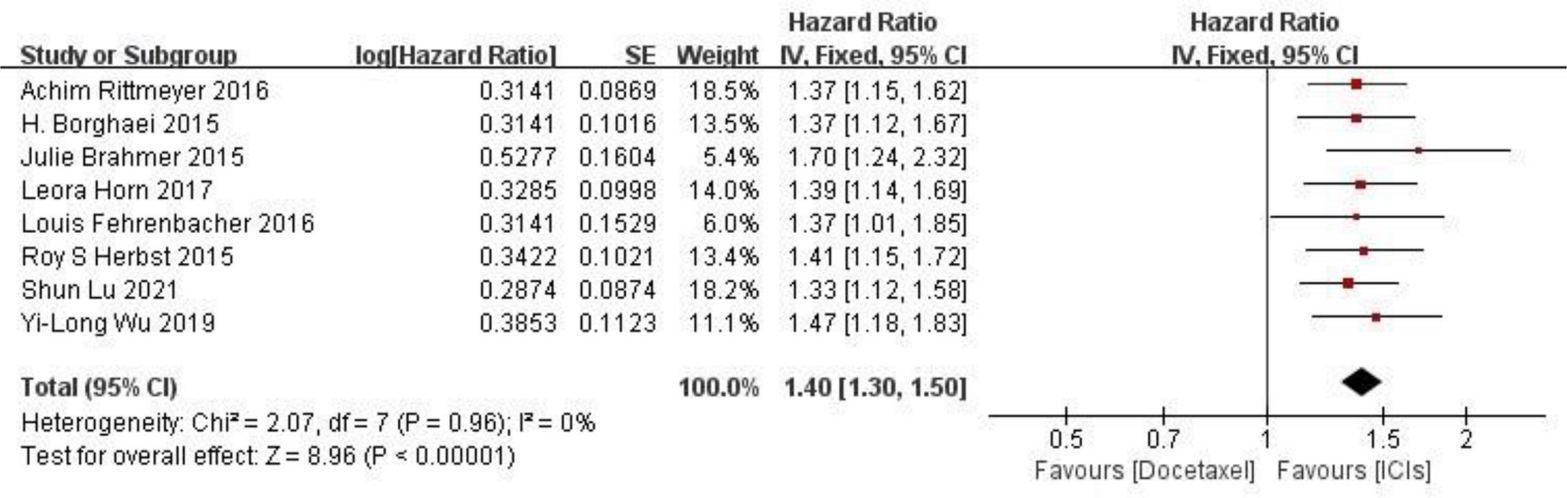
Meta-analysis results of OS between ICIs group and docetaxel group.

**OS comparison** 8 RCTs reported the OS of patients, and there were no statistically significant differences between studies. The results of meta-analysis showed that the OS of ICIS treatment group was longer than that of docetaxel chemotherapy group, and the difference was statistically significant (HR = 1.40, 95% CI: 1.30-1.50, P < 0.00001), indicating that the efficacy of ICIs in the treatment of NSCLC was better than that of docetaxel chemotherapy (as shown in **Figure 3**).

**Figure 4:**
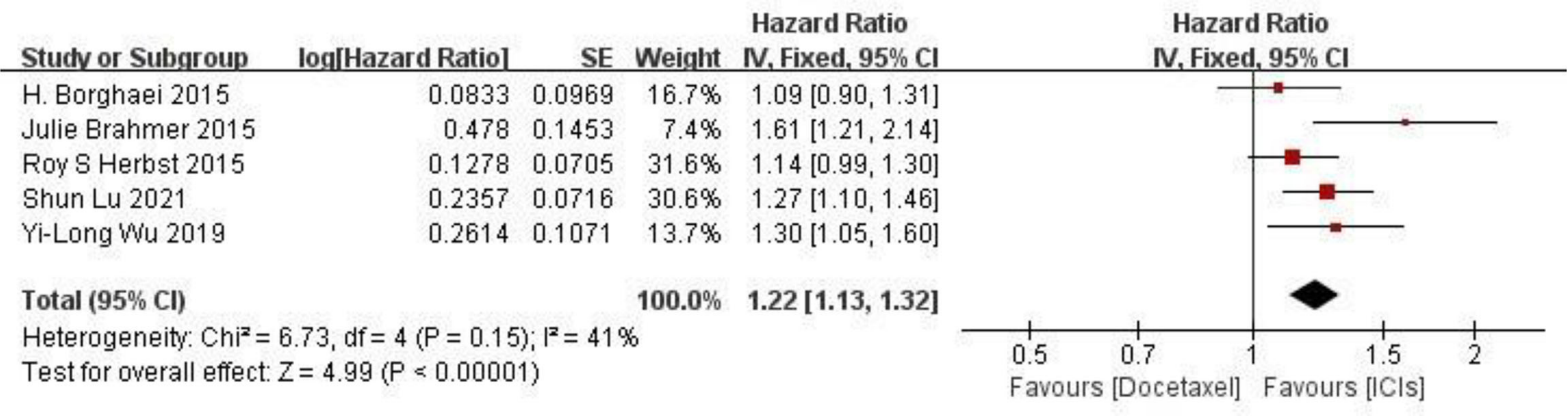
Meta-analysis results of PFS between ICIs group and docetaxel group.

**PFS comparison** Five RCTs reported PFS of patients, and there were no statistically significant differences between studies. The results of meta-analysis showed that the PFS of ICIS treatment group was longer than that of docetaxel chemotherapy group, and the difference was statistically significant (HR = 1.22, 95%CI: 1.13-1.32, P < 0.00001), indicating that the efficacy of ICIs in the treatment of NSCLC was better than that of docetaxel chemotherapy (as shown in **Figure 4**).

### Results of Safety Meta-Analysis of ICIs and Docetaxel

**Figure 5:**
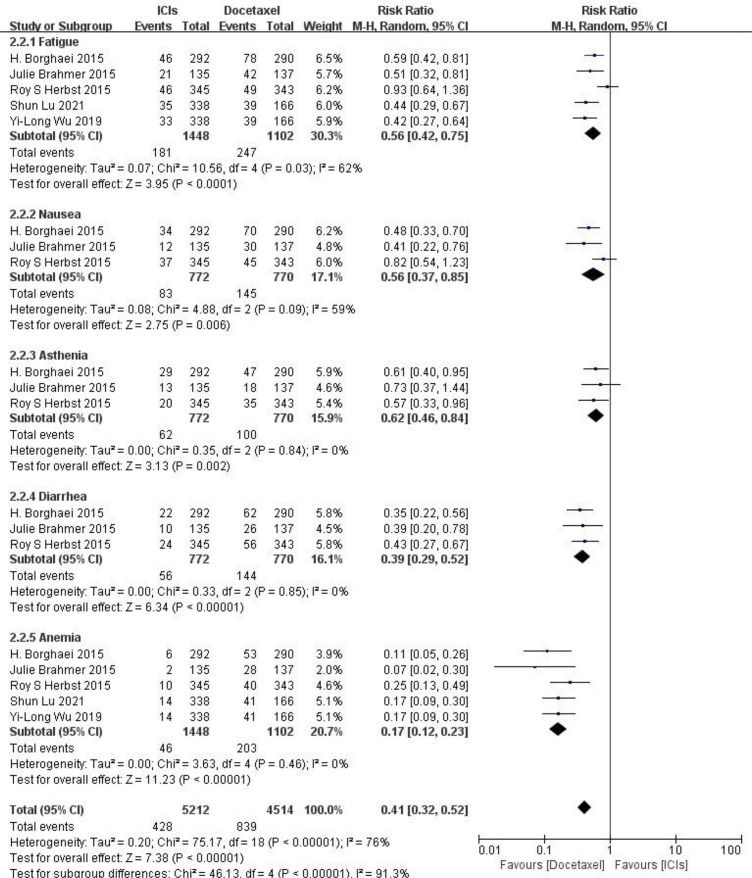
Meta-analysis results of adverse reactions of any grade between ICIs group and docetaxel group.

**Adverse reactions at any level** Five RCTs reported typical adverse reactions at any level (including fatigue, nausea, ashenia, diarrhea and anemia). There were no statistically significant differences between studies. The results of meta-analysis showed that the risk of adverse reactions at any level in the ICIs treatment group was lower than that in the docetaxel chemotherapy group, and the difference was statistically significant (HR = 0.41, 95%CI: 0.32-0.52, P < 0.00001), indicating that the safety of ICIs is superior to docetaxel monotherapy in NSCLC (as shown in **Figure 5**).

**Figure 6:**
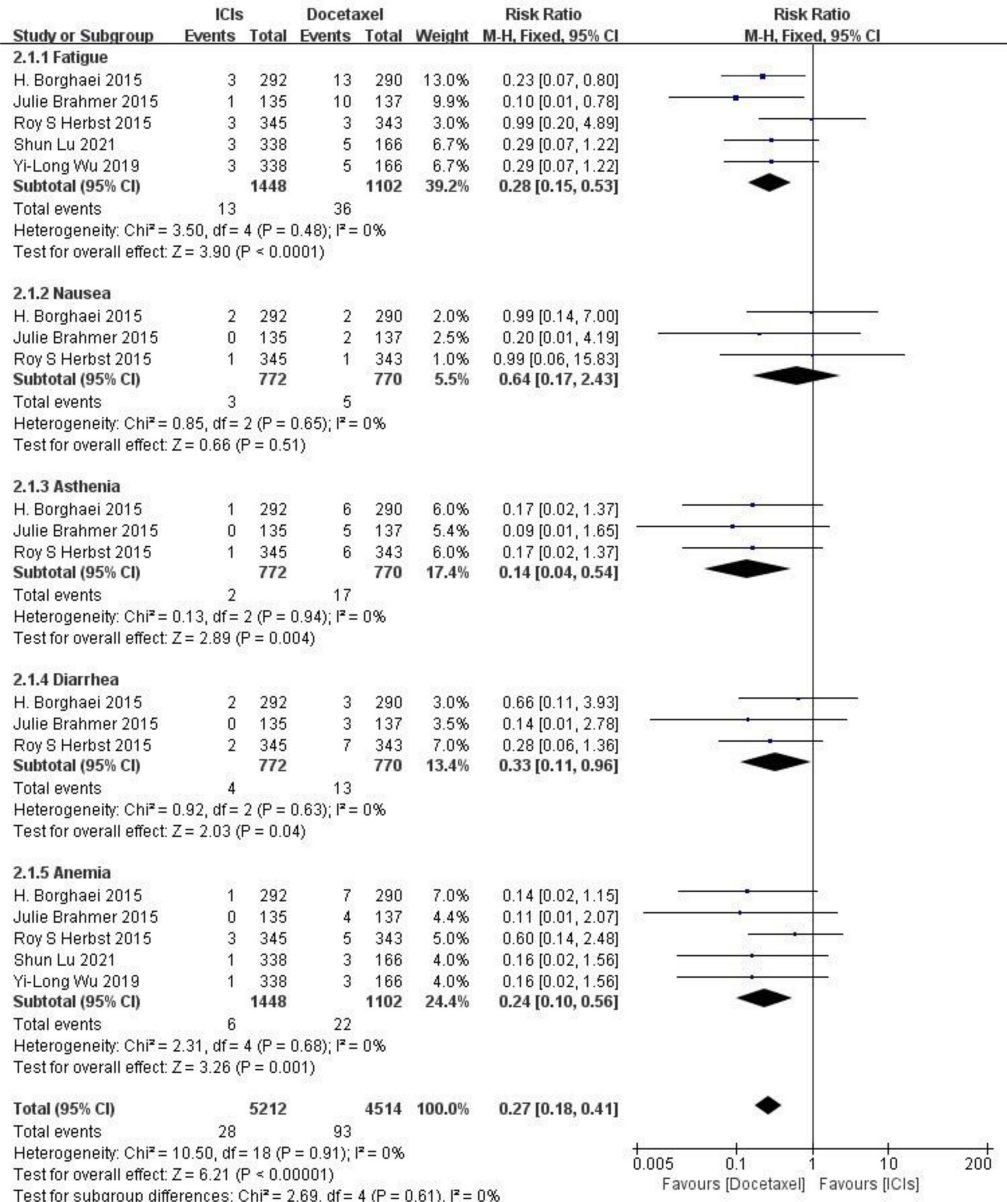
Meta-analysis results of adverse reactions above grade 3 between ICIs group and docetaxel group.

**Adverse reactions above grade III** Five RCTs reported typical adverse reactions above grade III (including fatigue, nausea, ashenia, diarrhea and anemia), and there were no statistically significant differences between studies. The results of meta-analysis showed that the risk of grade III and above adverse reactions in the ICIs treatment group was lower than that in the docetaxel chemotherapy group, and the difference was statistically significant (HR = 0.27, 95%CI: 0.18-0.41, P < 0.00001), indicating that the safety of ICIs is superior to docetaxel monotherapy in NSCLC(as shown in **Figure 6**).

### Sensitivity Analysis and Publication Bias Assessment

**Figure 7:**
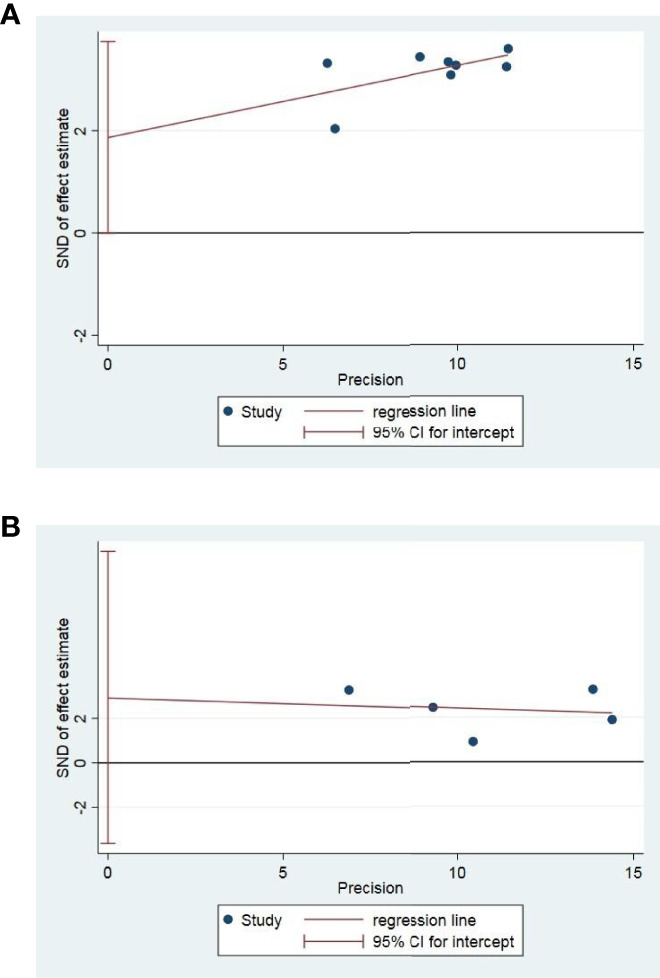
Egger test of OS **(A)** and PFS **(B)**.

Publication bias assessment was performed only in OS and PFS. Egger test in Stata12 software was used for publication bias test. In a total of 8 studies with OS and PFS as outcome indicators, the results of publication bias test indicated that there was no publication bias OS(P = 0.051) and PFS(P = 0.255), as shown in **Figure 7**.

## Discussion

In recent years, the choice of standard treatment for NSCLC patients has gradually changed from routine first-line drugs to immune checkpoint inhibitor (ICIs) therapy, or as a monotherapy, or combined with chemotherapy, anti angiogenic antibodies or other forms of ICIs. ICIs, an antibody against PD-1 or PD-L1, was approved for second-line and third-line treatment in patients with metastatic NSCLC without treatable driver mutations in 2015 (15). Since then, ICIs has been approved for first-line treatment, or for tumors with PD-L1 expression ≥ 50% alone, or in combination with chemotherapy independent of receptor status (16). Some patients treated with ICIs have particularly long-lasting response and survival. The study confirmed that up to 16% of patients with stage IV non-small cell lung cancer received second-line treatment with PD-1 inhibitor nivolumab and 31.9% received first-line treatment with PD-1 inhibitor pembrolizumab, with a survival time of 5 years (17). Another study showed that 4-year OS rates in POPLAR were 14.8% and 8.1% (and those in OAK were 15.5%and 8.7% for atezolizumab and docetaxel, respectively. However, it is worth noting that some patients with low PD-L1 expression may have poor efficacy in the treatment of tumors with immune checkpoint inhibitors. Therefore, it is very important to select biomarkers that can effectively predict the efficacy of PD-1/PD-L1 inhibitors, which is also an urgent problem to be solved in immunotherapy at this stage (18). At the same time, immune checkpoint inhibitors may cause immune related adverse reactions and infusion related reactions in the process of clinical application, which still needs further research (19).

In this meta-analysis, we evaluated the efficacy of ICIs drugs and docetaxel in patients with NSCLC, and selected OS and PFS as the primary outcomes. The results showed that ICIs improved the HR and *p* of OS and PFS in terms of effectiveness, indicating that patients receiving immunotherapy had better OS and PFS than patients receiving docetaxel. In terms of safety, the risk ratio of adverse reactions at any level and above in the ICIs treatment group was significantly lower than that in the docetaxel group, suggesting that the safety of ICIs treatment was higher than that of docetaxel, and it is not easy to produce common and typical adverse reactions (fatigue, nauesa, ashenia, diarrhea, anemia). Many international researches also show similar results, Khan M et al. (20) shows that compared with chemotherapy drugs, ICI therapy (nivolumab, pembrolizumab, atezolizumab) leads to better OS (HR 0.72 [95% CI 0.63, 0.82; P <. 00001]), PFS (HR 0.84 [95% CI 0.72), 0.97; P <. 02]) and ORR (odds ratio [OR] 1.52 [95% CI 1.08, 2.14; P <. 02]). At the same time, higher safety was observed with ICI therapy (OR 0.31 [95% CI 0.26, 0.38; P <. 00001]). The results of this study are basically consistent with those of previous international studies.

This study also has some limitations: ① after systematic retrieval and screening, only 8 literatures were included for systematic evaluation and meta-analysis, and the sample size is too small; ② The heterogeneity of individual statistical results may affect the credibility of the research results; ③ Different types of NSCLC in different studies may increase heterogeneity and affect the reliability of the results. However, in the study, in order to better reduce the above bias, when implementing retrieval and data consolidation, this study will report scientifically and objectively as much as possible in accordance with the Cochrane system evaluation guidance manual.

In conclusion, compared with docetaxel, ICIs can prolong the OS and PFS of patients with NSCLC, with better clinical efficacy. This therapy may be a promising treatment. However, it still needs to be further confirmed by studies with multiple centers, larger sample size and higher quality.

## Data Availability Statement

The original contributions presented in the study are included in the article/supplementary material. Further inquiries can be directed to the corresponding author.

## Author Contributions

WY and BX searched the database and analysed the data. MC, XL, JH and HS selected the study and extracted the data. WY and BX wrote the manuscript. YZ reviewed the manuscript. All authors contributed to the article and approved the submitted version.

## Funding

This work was supported by the clinical fund project of Zhejiang Medical Association in 2021 (2021ZYC-A193).

## Conflict of Interest

The authors declare that the research was conducted in the absence of any commercial or financial relationships that could be construed as a potential conflict of interest.

## Publisher’s Note

All claims expressed in this article are solely those of the authors and do not necessarily represent those of their affiliated organizations, or those of the publisher, the editors and the reviewers. Any product that may be evaluated in this article, or claim that may be made by its manufacturer, is not guaranteed or endorsed by the publisher.
